# Investigation of the Validity and Reliability of the Turkish Version of the Basic Activities of Daily Living Questionnaire in Preschool Children

**DOI:** 10.1155/oti/2729639

**Published:** 2025-01-10

**Authors:** Nilay Şahan, Songül Atasavun Uysal

**Affiliations:** ^1^Department of Occupational Therapy, Faculty of Health Sciences, Çankırı Karatekin University, Çankırı, Türkiye; ^2^Faculty of Physical Therapy and Rehabilitation, Hacettepe University, Ankara, Türkiye

**Keywords:** daily life, executive function, preschool children

## Abstract

Basic Activities of Daily Living-Preschool Period Tool **(**BADL-P) is a fast and quick test that does not require a special environment, where activities of daily living (ADLs) in preschool children are questioned. The aim of the study was to conduct a Turkish reliability and cultural adaptation of the BADL-P. The study was conducted among 211 parents of preschool children. Validity was determined using exploratory factor analysis. To determine convergent validity, the Functional Independence Measure for Kids (WeeFIM) scale was used. Cronbach's alpha, intraclass correlation coefficient (ICC), and item–total correlation (ITC) values were calculated to assess the reliability of the BADL-P. Totally, 99 girls and 112 boys, aged 3–6 years, with the children's mean age of 4.50 ± 0.75 years (3 years = 11%, 4 years = 32.1%, 5 years = 52.2%, and 6 years = 4.8%), were included. Exploratory factor analysis revealed that the BADL-P had sufficient fit. The BADL-P was weakly to highly correlated with the WeeFIM (*p* < 0.05 and *p* < 0.001). Cronbach's alpha and ICC values of the BADL-P were acceptable (0.912 and 0.837, respectively). The ITC values of each item of the BADL-P were also acceptable (ranged from 0.232 to 0.683). Children in the 5–6 age group demonstrated higher scores and exhibited more advanced development in the ADL areas compared to children in the 3–4 age group (*p* < 0.05). The Turkish version of the BADL-P is valid, reliable, and sensitive to assessing and monitoring the performance of Turkish preschool children in ADL.

## 1. Introduction

The basic activities of daily living (ADLs) that each person performs in order to be independent are defined as activities related to eating, dressing, hygiene, and self-care [1, 2]. The preschool period is a time of great development in the ADL [3]. This is because while the basic ADLs are carried out by young children, the variety and level of difficulty of these activities change with age [4].

The ability to perform ADLs is an important factor in children's development [5], because good performance in ADLs in the preschool period is important in terms of the individual's ability to achieve independent, self-sufficient, and social participation [3, 6]. Otherwise, problems in the realisation of ADLs may lead to unsafe conditions, overburdening the caregiver's responsibility for the child's daily living activities and low quality of life [3].

The realisation of appropriate ADLs depends on several factors. One of these factors is the maturation of the brain, in particular the perception, memory, and executive functions that are realised through the interaction of the prefrontal cortex [7, 8]. The World Health Organization defines executive functions as certain mental functions specifically associated with the frontal lobe of the brain, such as decision-making, abstract thinking, planning and execution, flexibility, and deciding which behaviours are appropriate in which circumstances [9, 10]. There are three main areas of executive function: working memory (ensuring that relevant information is retained during a task), inhibition (controlling attention, behaviour, and thoughts by suppressing other stimuli), and flexibility (describing the person's ability to change behaviour) [6]. These areas develop in different age groups throughout childhood and adolescence. Working memory and inhibition develop in the preschool years, while flexibility develops in the school years [10]. Therefore, appropriate executive functions are necessary for the ADLs in which we constantly plan and sequence, focus and organise, and solve problems [6].

It is noted that assessment scales such as the Pediatric Evaluation of Disability Inventory (PEDI) [11], the Functional Independence Measure for Kids (WeeFIM) [12], and the Activities Scales for Kids (ASK) [13] are used in the field to assess ADLs. In Türkiye, the PEDI and the WeeFIM, which are mostly valid and reliable, are used to assess and monitor Turkish children's performance in ADLs and to observe treatment outcomes [11]. The PEDI is a long scale consisting of 197 questions [11]. WeeFIM shows children's levels in ADLs but is said to be insensitive to interventions and changes over time. This makes it difficult to predict children's development [12].

Considering all these, it is seen that there is a need for cultural adaptation and reliability of the Basic Activities of Daily Living-Preschool Period Tool (BADL-P) in the assessment of ADLs in children. In addition to the basic ADL, the BADL-P also asks about executive functions that are important and necessary for the realisation of the ADL. The aim of the study was to conduct a Turkish reliability, validity, and cultural adaptation of the BADL-P and to add to the literature a scale that can be used by researchers working in this field to assess children's ADLs.

## 2. Materials and Methods

### 2.1. Participants

The study was conducted among 211 parents of preschool children living within the borders of Çankırı Province. The study included children with typical development, without any chronic diagnosis or history of disease, and not using any regular medication, who were attending any public preschool institution. Once the requisite permissions had been obtained from the Çankırı Provincial Directorate of National Education for the study, the relevant schools were contacted and the families of the participating children were informed. The study aims and procedures were explained to the children's families, and informed consent was obtained from parents. The study data were collected from seven preschool schools in Çankırı Province. For test–retest validity, the questionnaire was administered again after 2 weeks. The study was conducted with the approval of the Çankırı Karatekin University Ethics Committee in its meeting on 28 June 2022, Number 26, with the Verification Code c082e1a5c4704c89 and in accordance with the Declaration of Helsinki.

### 2.2. Instruments

The BADL-P was developed in Spanish by Barrios-Fernandez et al. to assess ADLs in preschool children aged 3–6 years [3]. The BADL-P consists of four different scales and a total of 84 questions, including three basic ADLs, namely, eating, personal hygiene, and dressing, and one daily functioning scale that assesses the cognitive functions necessary to perform basic ADLs.

The eating scale consists of a total of 16 questions under the subheadings of oral sensitivity while eating (three questions), good manners (seven questions), and manual dexterity while eating (six questions). The scale asks about sensory integration; behaviours of social, cultural, and educational origin that should be taken into account for good mealtime behaviour; and the manual skills required to use cutlery or serve food that are necessary for children to be able to feed themselves.

The personal hygiene scale consists of brushing teeth (six questions), sphincter control and management of needs in the water closet (WC) (nine questions), and hygiene and grooming (15 questions), for a total of 30 questions. This part of the scale is divided into three factors: all items related to tooth brushing, those related to toileting needs, and all activities related to personal hygiene and grooming. The concept of personal hygiene includes concepts such as providing and using toiletries, brushing teeth, bathing, and grooming to get and stay clean.

The dressing scale consists of a total of 21 questions. In this section, there are questions about choosing; wearing; and taking off clothes, shoes, and accessories. The scale has one factor.

The daily functioning scale consists of higher-order executive functions (eight questions) and core executive function (nine questions) subscales that affect the performance of ADLs and a total of 17 questions. The scale consists of two factors, high-level executive functions, and core executive functions.

The BADL-P is completed by interviewing the child's caregiver or parent. The caregiver or parent is asked to select one of four responses (3 = always, 2 = sometimes, 1 = never, and 0 = do not know/no way) according to the behaviour they observe in their child. The total score of the four subscales is calculated separately [3].

The WeeFIM is a scale based on the Adult Functional Independence Scale (FIM) used to assess functional independence in children. It is a scale consisting of a total of 18 items with six subheadings: self-care, sphincter control, mobility-transfers, locomotion, communication, and social perception. Each item is scored from 1 (completely assisted activities) to 7 (completely independent activities). The total score ranges from 18 to 126 [14].

#### 2.2.1. Turkish Translation, Validity, and Reliability of the BADL-P

In order to conduct the study, permission to translate the questionnaire into Turkish and use it in the research was obtained from the responsible author who developed the BADL-P in April 2022. In the Turkish translation and crosscultural adaptation of the BADL-P, which was originally written in Spanish, the methods and recommendations of Wild et al. [15] were employed. The questionnaire was initially translated into Turkish by two sworn translators who are native Spanish speakers. The two translations were then subjected to a comparison, after which the Turkish version of the questionnaire was created by two physiotherapists with field experience in ADL assessment and intervention in the clinical setting. Finally, the Turkish version of the questionnaire was translated into Spanish, with due consideration of the Turkish sociocultural structure. In the cultural adaptation of the BADL-P, “toilet management” was changed to “sphincter control and management of needs in the WC.” A questionnaire was administered to the families of 10 children. It was observed that there was no difficulty in understanding any question during the application.

### 2.3. Statistical Analysis

The study was completed using SPSS Statistics 26.0 to analyze the data. The data on the measurable variables was presented as the mean ± standard deviation (*x* ± SD), while the data on the categorical variables was presented as the number and percentage. Demographic features were taken, and the reliability and validity of the Turkish version of the BADL-P were examined. The Kaiser–Meyer–Olkin (KMO) and Bartlett's sphericity tests were used as indices of sampling adequacy [16]. The minimum acceptable KMO result was considered 0.5, and the results under 0.5 were accepted as nonfactorable dataset. Cronbach's alpha and the item–total correlation (ITC) values were calculated to determine internal consistency. To determine test–retest reliability, the intraclass correlation coefficients (ICCs) were calculated. Values of 0.70 for Cronbach's alpha, 0.50 for ICC, and 0.20 for ITC are considered minimum acceptable values [17, 18]. Differences were assessed as significant with an *α* value of 0.05. The model fitting indices including minimum discrepancy of confirmatory factor analysis/degrees of freedom (CMIN/df), goodness-of-fit index (GFI), comparative fit index (CFI), normed fit index (NFI), and root mean square error of approximation (RMSEA) statistics were used. These values were accepted as acceptable if the scores were between 0.05 and 1.0 for RMSEA and higher than 0.90 for fit indices [19]. Normality distribution was tested with the Kolmogorov–Smirnov test, and the data showed a normal distribution. The Pearson correlations between the BADL-P and the WeeFIM performance level were calculated to determine the convergent validity. In order to ascertain the scale's sensitivity, the difference between the groups in terms of gender and age was measured using a two-sample *t*-test.

## 3. Results

### 3.1. Demographic Characteristics of Individuals

A total of 211 children, comprising 99 girls and 112 boys, were administered the BADL-P and WeeFIM. In the study conducted with the parents of children aged 3–6 years, the mean age of the children was 4.50 ± .75 years (3 years = 11%, 4 years = 32.1%, 5 years = 52.2%, and 6 years = 4.8%). The mean body mass index (BMI) of the children in the study was 15.91 ± 2.28 kg/m^2^. The mean age of the mothers of the children was 31.54 ± 5.07 years, and the mean age of the fathers was 35.02 ± 5.10 years. The majority of mothers (43.9%) and fathers (33.1%) had obtained a university education. The demographic data of the participants is presented in Table 1.

### 3.2. Construct Validity

The construct validity of the Turkish version of the BADL-P was tested with the confirmatory factor analysis (CFA). The calculated fit index values for the eating scale were as follows: *X*^2^/df = 1.327, *p* = 0.017, GFI = 0.928, CFI = 0.953, NFI = 0.938, and RMSEA = 0.039; for the personal hygiene scale *X*^2^/df = 2.081, *p* = 0.001, GFI = 0.892, CFI = 0.898, NFI = 0.890, and RMSEA = 0.069; for the dressing scale *X*^2^/df = 2.075, *p* = 0.001, GFI = 0.898, CFI = 0.894, NFI = 0.926, and RMSEA = 0.069; and for the daily functioning scale *X*^2^/df = 1.765, *p* = 0.001, GFI = 0.899, CFI = 0.901, NFI = 0.901, and RMSEA = 0.060. According to the CFA statistics, the Turkish version of the BADL-P demonstrated an acceptable fit (Table 2).

For the eating scale, the KMO value of 0.700 and Bartlett's test, *p* < 0.001; for the personal hygiene scale, the KMO value of 0.785 and Bartlett's test, *p* < 0.001; for the dressing scale, the KMO value of 0.866 and Bartlett's test, *p* < 0.001; and for the daily functioning scale, the KMO value of 0.825, and Bartlett's test, *p* < 0.001, were all good enough to carry out the exploratory factor analysis.

### 3.3. Convergent Validity

A statistically significant correlation was identified between the subheadings of the BADL-P and the subheadings of WeeFIM, with correlations ranging from weak to high (*r* = −0.415–0.836; *p* < 0.05 and *p* < 0.001).

### 3.4. Reliability

Cronbach's *α* coefficient value of the Turkish version of the BADL-P questionnaire was found to be excellent, with a value of 0.912 for the overall questionnaire. The reliability levels of the subheadings of the questionnaire were found to be satisfactory for the subheadings of oral sensitivity (0.700), good manners (0.703), manual dexterity while eating (0.759), brushing teeth (0.740), sphincter control and management of needs in the WC (0.695), and higher-order executive functions (0.797). Cronbach's *α* coefficient value of the Turkish version of the BADL-P questionnaire was found to be excellent for the following subheadings: personal hygiene (0.819) and dressing (0.901) and core executive function (0.805). It was observed that the results of the correlation between the items and the total ranged from 0.232 to 0.683 (Table 3).

### 3.5. Test–Retest Reliability

The test–retest ICC values of the Turkish version of the BADL-P questionnaire were found to be between 0.837 and 0.963. In the subheadings of the questionnaire, the highest test–retest ICC values were found in the eating subheading (0.963) and the lowest in the daily functioning subheading (0.837). The interval between the two tests was 2 weeks.

### 3.6. Scale Response Bias

In the study, the Hotelling *T*^2^ test was employed to assess whether the responses of the individuals to the scale items were equivalent. The results of this test indicated that Hotelling *T*^2^ = 9840.791 and *p* < 0.001 for the BADL-P. This demonstrated that there was no response bias in the scale.

### 3.7. Sensitivity

A comparison of the children who participated in the BADL-P according to age groups revealed a statistically significant difference between the two groups, with the exception of the subitem of sphincter control and management of needs in the WC. As illustrated in Table 4, children in the 5–6 age group demonstrated higher scores and exhibited more advanced development in the ADL areas compared to children in the 3–4 age group (Table 5).

A statistical analysis of the data revealed that girls participating in the study exhibited statistically higher scores than boys in the personal hygiene scale and dressing scales (Table 6).

## 4. Discussion

As a result of the study, the BADL-P Turkish was found to be valid and reliable for assessing and monitoring the performance of Turkish preschool children in ADL and for observing treatment outcomes, and cultural adaptations were made.

The preschool years are the first 6 years of a child's life. This period is the most important years of a child's life, during which the child's development is very rapid, the child's character and personality structure begin to develop, and the child acquires basic skills for daily activities. These acquired basic daily living activities are behaviours that ensure the child's adaptation to and participation in the environment and society in which they live. Once these behaviours are learned, they are repeated throughout the day and continue to exist throughout life [4, 20].

Toilet training is one of the most fundamental and essential life skills that children should be able to achieve. The age of the child; their linguistic abilities; their physical and mental development; and a number of other factors, including cultural differences, sociodemographic characteristics, and the educational level of the child's parents, can all influence the effectiveness of this training [21]. In Türkiye, children are required to complete toilet training in order to be eligible for admission to preschool education institutions. Furthermore, studies have indicated that toilet training is initiated at an earlier age in Türkiye and completed between 2 and 2.5 years of age [21, 22]. As reported in the original article by Barrios-Fernandez et al., in which the questionnaire was developed in Spanish, it was observed that Turkish children achieved higher scores than Spanish children [3]. Furthermore, the reliability of the toilet management subheading of the Turkish BADL-P was found to be inferior to that of the other subheadings. This result may be due to the fact that children may have learnt the activities under this heading at an earlier age because toilet training in Turkish culture starts and is completed at an earlier age and families do not report problems in this regard. In addition, the fact that we conducted the study in children with typical development may have been another factor.

Furthermore, the test–retest reliability of the study is satisfactory. Additionally, the results of the analyses indicate that there was no response bias in answering the scales. These findings collectively demonstrate the consistency of the study.

In the study, children were divided into two groups according to their ages, with the first group comprising children aged 3–4 years and the second group comprising children aged 5–6 years. The findings indicated that children in the 5–6 age group outperformed children in the 3–4 age group in all areas of daily living, as predicted. Although this situation emerged as predicted, it was thought that it might be due to the fact that all children participating in the study were typically developing children, all of whom attended preschool education institutions [20, 23], and their parents' education levels were high [24].

In examining the status of children in ADLs in terms of gender, Barrios-Fernandez et al. found that girls exhibited superior performance in the sphincter control and management of needs in the WC subheadings of the personal hygiene scale and in the core executive function subheadings of the daily functioning scale in the BADL-P. In this study, results indicated that girls outperformed boys in all subheadings of the personal hygiene scale and in the dressing scale. It is postulated that this phenomenon can be attributed to the cultural structure of Turkish society.

During the Turkish adaptation of the BADL-P, a number of observations were made. One such observation was the question in the manual dexterity during eating subheading of the eating scale: “uses a knife to cut food.” Parents, particularly those with children in the younger age group, stated that they did not allow their children to use the knife because they believed it to be dangerous and cutting would harm themselves. Another situation encountered in the application was that the majority of parents stated that they intervened with their child in the question “cleans himself/herself in an acceptable way with toilet paper” in the control of sphincters and management of needs in the WC subheading and in the washing/bathing questions in the hygiene and grooming subheading. In the dressing scale, another noteworthy observation was that parents of younger children, in particular, expressed a preference for shoes without laces. This preference was reflected in the children's inability to tie their own shoes. It is postulated that these parental interventions may be attributed to the more controlling and protective parental model that is prevalent in the Turkish cultural structure.

The BADL-P is a fast and quick test that does not require a special environment, where ADLs in preschool children are questioned by the therapist and parent/caregiver interview. In addition, other advantages are that it includes activities reflecting the physiological conditions of children that change with age and that it can measure the ADL in a wide variety of areas such as oral sensitivity, eating rules, and manual skills required for nutrition. The BADL-P differs from other scales in that it measures the fundamental ADLs and the executive functions necessary for the realisation of ADLs. The most significant attribute of the BADL-P is that the scales that comprise the BADL-P can be employed independently, thereby facilitating the assessment of the components deemed necessary by the expert.

The Turkish version of the BADL-P was found to be readily comprehensible and straightforward to administer. It is postulated that the high level of education among the families may have contributed to the ease of comprehension. Nevertheless, it is believed that conducting ADL assessments of preschool children is highly beneficial, with the therapist observing the child performing the activity in a natural or simulated environment and obtaining information from the family being two key methods of assessment. In particular, it is of great importance to educate, guide, and inform the families of children of this age, in order that they may assist their children in becoming independent individuals. This can be achieved by performing the necessary ADL activities in accordance with their age.

The study has a limitation that has to be taken into account that the age and gender groups of the children participating in the study were not equal.

The next study will examine the validity and reliability of this questionnaire in children with any disability.

The BADL-P is an accessible and dependable instrument for the evaluation and monitoring of preschool Turkish children's performance in ADLs. It is believed to be a valuable tool for therapists engaged in this field.

## Figures and Tables

**Table 1:** Demographic information of children.

**N** = 211	**Categories**	**n** ** (%)**	**M** **e** **a** **n** ± **SD**
Gender	Female	99 (46.9%)	
	Male	112 (53.1%)	
Age (year)			4.50 ± 0.75
BMI (kg/m^2)^			15.91 ± 2.28
Mother's age (year)			31.54 ± 5.07
Mother's education	Primary school	15 (7.3%)	
	Middle school	37 (18%)	
	High school	63 (30.7%)	
	University	90 (43.9%)	
Father's age (year)			35.02 ± 5.10
Father's education	Primary school	4 (1.4%)	
	Middle school	24 (8.6%)	
	High school	83 (29.9%)	
	University	92 (33.1%)	

**Table 2:** BADL-P goodness-of-fit indices.

		**Eating scale**	**Personal hygiene scale**	**Dressing scale**	**Daily functioning scale**
Chi-squared probability *p* (*X*^2^)	> 0.05	0.017	0.000	0.000	0.000
GFI	> 0.90	0.928	0.892	0.898	0.899
CFI	> 0.90	0.953	0.898	0.894	0.901
RMSEA	< 0.80	0.039	0.069	0.069	0.060
NFI	> 0.90	0.938	0.890	0.926	0.901

**Table 3:** Internal consistency (item–total correlations and Cronbach's *α* coefficients) of the Turkish version of BADL-P.

**N** = 211	**Median (min–max)**	**M** **e** **a** **n** ± **SD**	**Item**–**total correlation**	**Item-deleted Cronbach's ** **α**	**Cronbach's ** **α**
*Eating scale*	25–47	36.34 ± 4.39			
Oral sensitivity while eating	6 (3–9)	5.44 ± 1.53			0.700
Oral Sensitivity While Eating 1	2 (1–3)	1.98 ± 0.62	0.517	0.609	
Oral Sensitivity While Eating 2	2 (0–3)	1.75 ± 0.65	0.585	0.521	
Oral Sensitivity While Eating 3	3 (0–3)	1.71 ± 0.66	0.453	0.690	
Good manners	19 (11–21)	19.03 ± 1.96			0.703
Good Manners 1	3 (0–3)	2.72 ± 0.47	0.293	0.700	
Good Manners 2	3 (2–3)	2.85 ± 0.35	0.324	0.690	
Good Manners 3	3 (0–3)	2.85 ± 0.36	0.373	0.680	
Good Manners 4	3 (1–3)	2.68 ± 0.49	0.519	0.639	
Good Manners 5	3 (1–3)	2.58 ± 0.54	0.353	0.689	
Good Manners 6	3 (1–3)	2.74 ± 0.48	0.472	0.653	
Good Manners 7	3 (1–3)	2.58 ± 0.51	0.571	0.622	
Manual dexterity while eating	12 (4–19)	11.91 ± 3.53			0.759
Manual Dexterity While Eating 1	3 (1–3)	2.56 ± 0.53	0.277	0.675	
Manual Dexterity While Eating 2	2 (0–3)	1.72 ± 0.91	0.475	0.731	
Manual Dexterity While Eating 3	2 (0–3)	1.99 ± 0.90	0.600	0.700	
Manual Dexterity While Eating 4	2 (0–3)	1.78 ± 1.00	0.683	0.675	
Manual Dexterity While Eating 5	2 (0–3)	1.82 ± 0.98	0.546	0.726	
Manual Dexterity While Eating 6	2 (0–3)	2.01 ± 0.82	0.415	0.736	
*Personal hygiene scale*	82 (50–90)	79.41 ± 8.03			
Brushing teeth	15 (3–18)	14.96 ± 2.45			0.740
Brushing Teeth 1	2 (0–3)	2.02 ± 0.65	0.459	0.709	
Brushing Teeth 2	3 (0–3)	2.51 ± 0.61	0.518	0.692	
Brushing Teeth 3	3 (0–3)	2.55 ± 0.59	0.526	0.690	
Brushing Teeth 4	3 (0–3)	2.83 ± 0.41	0.385	0.729	
Brushing Teeth 5	3 (0–3)	2.58 ± 0.67	0.452	0.712	
Brushing Teeth 6	3 (0–3)	2.44 ± 0.71	0.544	0.684	
Sphincter control and management of needs in the WC	26 (15–27)	25.20 ± 2.07			0.695
Sphincter Control and Management of Needs in the WC 1	3 (1–3)	2.85 ± 0.42	0.232	0.681	
Sphincter Control and Management of Needs in the WC 2	3 (1–3)	2.77 ± 0.48	0.340	0.676	
Sphincter Control and Management of Needs in the WC 3	3 (1–3)	2.93 ± 0.26	0.484	0.661	
Sphincter Control and Management of Needs in the WC 4	3 (1–3)	2.88 ± 0.31	0.552	0.644	
Sphincter Control and Management of Needs in the WC 5	3 (2–3)	2.94 ± 0.23	0.550	0.658	
Sphincter Control and Management of Needs in the WC 6	3 (0–3)	2.53 ± 0.63	0.369	0.681	
Sphincter Control and Management of Needs in the WC 7	3 (1–3)	2.87 ± 0.32	0.516	0.648	
Sphincter Control and Management of Needs in the WC 8	2 (1–3)	2.82 ± 0.40	0.504	0.643	
Sphincter Control and Management of Needs in the WC 9	3 (0–3)	2.57 ± 0.58	0.283	0.690	
Hygiene and grooming	41 (21–45)	39.69 ± 4.94			0.819
Hygiene and Grooming 1	3 (0–3)	2.60 ± 0.54	0.326	0.819	
Hygiene and Grooming 2	3 (0–3)	2.73 ± 0.48	0.335	0.814	
Hygiene and Grooming 3	3 (0–3)	2.62 ± 0.59	0.414	0.810	
Hygiene and Grooming 4	3 (0–3)	2.70 ± 0.53	0.315	0.815	
Hygiene and Grooming 5	3 (1–3)	2.78 ± 0.42	0.396	0.820	
Hygiene and Grooming 6	3 (0–3)	2.84 ± 0.41	0.358	0.817	
Hygiene and Grooming 7	3 (0–3)	2.47 ± 0.74	0.415	0.810	
Hygiene and Grooming 8	3 (0–3)	2.89 ± 0.34	0.357	0.814	
Hygiene and Grooming 9	3 (1–3)	2.86 ± 0.35	0.465	0.810	
Hygiene and Grooming 10	3 (1–3)	2.72 ± 0.46	0.371	0.812	
Hygiene and Grooming 11	3 (1–3)	2.82 ± 0.42	0.514	0.806	
Hygiene and Grooming 12	3 (0–3)	2.38 ± 0.86	0.631	0.792	
Hygiene and Grooming 13	3 (0–3)	2.49 ± 0.86	0.639	0.791	
Hygiene and Grooming 14	3 (0–3)	2.54 ± 0.77	0.655	0.790	
Hygiene and Grooming 15	3 (0–3)	2.22 ± 0.97	0.654	0.791	
*Dressing scale*	54 (24–63)	52.20 ± 7.92			0.901
Dressing 1	3 (0–3)	2.50 ± 0.69	0.438	0.899	
Dressing 2	3 (1–3)	2.74 ± 0.45	0.537	0.897	
Dressing 3	3 (1–3)	2.91 ± 0.29	0.475	0.899	
Dressing 4	3 (1–3)	2.72 ± 0.46	0.362	0.900	
Dressing 5	3 (0–3)	2.52 ± 0.60	0.456	0.898	
Dressing 6	3 (1–3)	2.93 ± 0.27	0.320	0.901	
Dressing 7	3 (1–3)	2.66 ± 0.53	0.525	0.897	
Dressing 8	2 (0–3)	2.28 ± 0.67	0.499	0.897	
Dressing 9	3 (0–3)	2.76 ± 0.47	0.434	0.899	
Dressing 10	3 (0–3)	2.75 ± 0.49	0.531	0.897	
Dressing 11	3 (0–3)	2.65 ± 0.58	0.532	0.897	
Dressing 12	3 (1–3)	2.68 ± 0.55	0.575	0.896	
Dressing 13	3 (0–3)	2.53 ± 0.69	0.538	0.896	
Dressing 14	3 (0–3)	2.58 ± 0.64	0.558	0.896	
Dressing 15	2 (0–3)	2.30 ± 0.78	0.624	0.894	
Dressing 16	3 (0–3)	2.53 ± 0.67	0.665	0.893	
Dressing 17	2 (0–3)	2.30 ± 0.77	0.624	0.894	
Dressing 18	2 (0–3)	2.11 ± 0.92	0.571	0.896	
Dressing 19	2 (0–3)	1.55 ± 0.91	0.595	0.895	
Dressing 20	2 (0–3)	1.61 ± 0.94	0.578	0.896	
Dressing 21	3 (0–3)	2.45 ± 0.68	0.668	0.892	
*Daily functioning scale*	35 (21–46)	34.52 ± 3.71			
High-order executive functions	21 (7–24)	20.32 ± 2.94			0.797
High-Order Executive Functions 1	3 (1–3)	2.75 ± 0.45	0.381	0.791	
High-Order Executive Functions 2	2 (0–3)	2.37 ± 0.63	0.508	0.774	
High-Order Executive Functions 3	2 (0–3)	2.32 ± 0.64	0.490	0.778	
High-Order Executive Functions 4	3 (1–3)	2.56 ± 0.51	0.475	0.779	
High-Order Executive Functions 5	3 (0–3)	2.52 ± 0.62	0.517	0.773	
High-Order Executive Functions 6	3 (0–3)	2.58 ± 0.61	0.629	0.753	
High-Order Executive Functions 7	2 (0–3)	2.41 ± 0.59	0.556	0.766	
High-Order Executive Functions 8	3 (0–3)	2.78 ± 0.47	0.495	0.777	
Core executive function	14 (7–24)	14.00 ± 3.47			0.805
Core Executive Function 1	2 (0–3)	2.05 ± 0.62	0.431	0.794	
Core Executive Function 2	1 (0–3)	1.40 ± 0.59	0.520	0.783	
Core Executive Function 3	1 (0–3)	1.49 ± 0.61	0.651	0.766	
Core Executive Function 4	1 (0–3)	1.46 ± 0.64	0.450	0.792	
Core Executive Function 5	2 (0–3)	1.72 ± 0.68	0.513	0.784	
Core Executive Function 6	2 (0–3)	1.56 ± 0.59	0.404	0.797	
Core Executive Function 7	1 (0–3)	1.46 ± 0.58	0.478	0.788	
Core Executive Function 8	1 (0–3)	1.31 ± 0.53	0.537	0.782	
Core Executive Function 9	1 (0–3)	1.51 ± 0.66	0.513	0.784	
*General total*					0.912

**Table 4:** Convergent–divergent validity of the Turkish version of the BADL-P and WeeFIM.

		**WeeFIM**								
		**Self-care**						**Sphincter control**	**Communication**	**Social perception**
		**Eating** ***r* (*p*)**	**Grooming, brushing teeth** ***r* (*p*)**	**Bathing** ***r* (*p*)**	**Dressing upper body** ***r* (*p*)**	**Dressing lower body** ***r* (*p*)**	**Toileting** ***r* (*p*)**	***r* (*p*)**	***r* (*p*)**	***r* (*p*)**
BADL-P	Eating scale *r* (*p*)	0.836⁣^∗∗^ (0.001)								
	Brushing teeth *r* (*p*)		0.730⁣^∗^ (0.001)							
	Sphincter control and management of needs in the WC *r* (p)						0.700⁣^∗∗^ (0.001)	0.414⁣^∗∗^ (0.001)		
	Hygiene and grooming *r* (*p*)		0.657⁣^∗^ (0.001)	0.724⁣^∗∗^ (0.001)						
	Dressing *r* (*p*)				0.721⁣^∗^ (0.001)	0.765⁣^∗∗^ (0.001)				
	High-order executive function *r* (*p*)								0.295⁣^∗∗^ (0.001)	0.791⁣^∗∗^ (0.001)
	Core executive function *r* (*p*)								−0.415⁣^∗∗^ (0.001)	−0.232⁣^∗∗^ (0.001)

*Note:r*: Pearson correlation test, *p*: statistical signification.

⁣^∗^<0.05.

⁣^∗∗^<0.001.

**Table 5:** Descriptive and contrasting statistics by age group factors.

**Years**	**Eating scale**			**Personal hygiene scale**			**Dressing scale**	**Daily functioning scale**	
	**Oral sensitivity**	**Good manners**	**Manual dexterity**	**Brushing teeth**	**Sphincter control and management of needs in the WC**	**Hygiene and grooming**	**Dressing**	**Higher-order EF factor**	**Core EF factor**
3–4 (*n* = 90)	5.72 ± 1.39	18.31 ± 1.98	10.70 ± 3.22	14.07 ± 2.78	24.94 ± 1.93	38.27 ± 5.02	48.30 ± 8.34	19.31 ± 2.87	14.57 ± 3.54
5–6 (*n* = 121)	5.24 ± 1.56	19.35 ± 1.89	12.84 ± 3.55	15.52 ± 2.25	25.35 ± 2.22	40.79 ± 4.50	55.00 ± 6.42	21.10 ± 2.80	13.64 ± 3.30
*t*	2.328	−3.826	−4.564	−4.041	−1.417	−3.751	−6.328	−4.503	1.937
*p*	0.021⁣^∗^	0.001⁣^∗∗^	0.001⁣^∗∗^	0.001⁣^∗∗^	0.066	0.001⁣^∗∗^	0.001⁣^∗∗^	0.001⁣^∗∗^	0.054⁣^∗^
*d*	0.32	0.54	0.63	0.58	0.19	0.53	0.92	0.63	0.36

*Note:t*: two-sample *t*-tests; *p*: statistical signification; *d*: Cohen's *d* (small = 0.2; medium = 0.5; large = 0.8).

⁣^∗^<0.05.

⁣^∗∗^<0.001.

**Table 6:** Descriptive and contrasting statistics by gender group factors.

**Gender**	**Eating scale**			**Personal hygiene scale**			**Dressing scale**	**Daily functioning scale**	
	**Oral sensitivity**	**Good manners**	**Manual dexterity**	**Brushing teeth**	**Sphincter control and management of needs in the WC**	**Hygiene and grooming**	**Dressing**	**Higher-order EF factor**	**Core EF factor**
Female (*n* = 99)	5.47 ± 1.51	19.05 ± 1.99	12.34 ± 3.56	15.42 ± 2.65	25.47 ± 1.98	40.91 ± 4.27	53.61 ± 7.77	20.67 ± 2.73	14.09 ± 3.63
Male (*n* = 112)	5.38 ± 1.53	18.78 ± 2.00	11.61 ± 3.57	14.47 ± 2.45	24.82 ± 2.28	38.72 ± 5.16	50.90 ± 8.04	20.03 ± 3.13	14.02 ± 3.32
*t*	0.432	0.961	1.478	2.691	2.226	3.375	2.490	1.586	0.133
*p*	0.666	0.337	0.141	0.008⁣^∗∗^	0.027⁣^∗^	0.001⁣^∗∗^	0.014⁣^∗^	0.114	0.894
*d*				0.37	0.25	0.46	0.34		

*Note:t*: two-sample *t*-tests; *p*: statistical signification; *d*: Cohen's *d* (small = 0.2, medium = 0.5, and large = 0.8).

⁣^∗^<0.05.

⁣^∗∗^<0.001.

## Data Availability

The data that support the findings of this study are available upon reasonable request but are not publicly accessible due to privacy or confidentiality concerns.
